# Treatment of Fracture of the Patella, Treated by the Open Method

**Published:** 1909-12

**Authors:** 


### Treatment of Fracture of the Patella, Treated by the Open Method

AIMÉ PAUL HEINECK, Chicago, *Surgery Gynecology and Obstetrics,* August, 1909, tabulates and analyses 1,100 fractures of the patella treated by the open operative method. He discusses the following questions:

1. Is the patella essential to the functional integrity of the the knee-joint?

2. Are permanent displacements of the patella, in whole or in part, congenital or acquired, or the rudimentary development of this bone, deformities significantly impairing the functions of the knee-joint?

3. Are there other traumatic lesions, simulating from the symptomatic standpoint, by the functional disturbances which they entail, fractures of the patella? What are these conditions? How are they best treated?

4. Which is the treatment of choice for fractures of the patella?

5. Is operation at times, contraindicated? If so, when?

6. If operation is not always indicated, when is it indicated?

7. How should the treatment of old fractures of the patella differ from that of recent fractures, or is the same treatment applicable to both? If not, why not?

8. Which of the principal various open operative procedures that are now in vogue for the treatment of fractures of the patella the most universally applicable, the most satisfactory from the standpoint of early and of late results: transverse or longitudinal osseous suturing, looping of the patella (cerclage Berger), hemi-cerclage (Quénu), or suturing of the peri- and para-patellar fibrous tissues; suture des ailerons, Vallas, retinaculæ patella, B. N. A., reserve extensor apparatus (Mikulicz).

9. Should one, if he be an advocate of the open operative treatment, operate on the day and on the morrow of the infliction of the injury, or should he wait till the soft tissues have recovered from the immediate effects of the traumatism?

10. Should the operative field be rendered bloodless by the employment of an Esmarch bandage?

11. What should be the nature of the anesthetic employed: local, lumbar, or general anesthesia?

12. By what type of incision is the operator best enabled to perform the repair work which he deems appropriate and necessary?

13. Is it advisable in these cases to irrigate the articulation; if so, with what fluid, an antiseptic solution, irritating or non-irritating, or merely a cleansing agent, such as normal salt solution? Or is the mere sponging out of the extravasated liquid and clotted blood, from the synovial cavity, productive of the most satisfactory results?

14. Should non-absorbable, or absorbable, suture material be used? Are there any valid reasons for discarding non-absorbable suture material?

15. Shall the periarticular tissues be drained?

16. Shall the articulation be drained?

17. Shall the completely detached bony fragments be removed?

18. What should be the duration and the nature of the post-operative treatment?

Heineck answers them as follows:

1. A careful study of the literature amply justifies the statement that congenital or acquired absence, unilateral or bilateral, of the patella, is always associated with some impairment of the functional integrity of the anatomically defective knee-joint or joints. This impairment in some cases is very slight; in other cases, it is considerable.

2. Any dislocation of the patella, be it intermittent or permanent, be it complete or incomplete, be it congenital or acquired, is also always associated with some impairment, slight or severe, of the functional integrity of the knee-joint.

3. All upward or downward displacements of the patella as a whole, if dependent upon rupture of the quadriceps extensor femoris tendon, or of the ligamentum patella, will cause symptoms somewhat analogous to those which are caused by complete transverse, oblique, stellate, or comminuted fractures of the patella. Violence of the same nature can determine a solution of continuity of either the tendon, the patella, or the ligament.

4. To secure the best functional results, it is essential that in fractures of the patella, the torn prepatellar fibroperiosteal tissues be carefully sutured. All tears in the parapatellar tissues must be sewed up. To contribute to the maintenance in opposition of the fragments, the patella is circumferentially looped by a ligature passed close to its periphery. This ligature is passed so as to be close to the periphery of the bone, so as to hug it, as it were. It is inserted in such a way that it lies imbedded in the substance of both quadriceps tendon and ligamentum patella, midway between their anterior and posterior surfaces. If deemed necessary, two such looping ligatures may be used. These different maneuvers are all extraärticular.

1. We do not advise the open operation.

(a) In fractures of the patella that occur in a diabetic patient, the tissues of diabetics offer very little resistance to infection.

(b) In fracture of the patella occurring in patients having advanced tubercular disease.

(c) In fractures of the patella occurring in patients suffering from well developed cardiac, renal, or hepatic disease, or the subject of the malignant disease.

2. In closed longitudinal fractures with no displacement, or with but slight lateral displacement.

3. In subaponeurotic or in incomplete fractures.

4. Fractures of the patella in which the separation of the patellar fragments is so slight as to be barely detectable do not call for the open operative treatment.

It is our belief that, after ample preparation of the patient and of the operative field, the open operative treatment is positively indicated:

1. In all fresh fractures of the patella in the absence of contraindications:

(a) If the surroundings are favorable.

1. An aseptic operating room.

2. Skilled surgeon, and assistants having "an aseptic conscience."

3. Dependable suture material, rubber gloves, and the like. contraindications:

(b) If the patient is in the best possible condition.

(c) If the fracture be of such a nature that a disabling defect is to be expected, if one resorts to nonoperative treatment.

(d) When the bony fragments cannot be returned exactly by manipulation to their normal position and retained therein by retentive apparatus.

2. In all compound fractures.

3. In all comminuted fractures.

4. In all cases associated with considerable intraärticular effusion.

5. In all cases associated with marked laceration of the periarticular tissues (ailerons, reserve extensor apparatus).

6. In all cases in which the interfragmentary space or diastasis has at any time exceeded 3 cm.

7. In such fractures as are very liable to cause serious functional joint impairment; among such may be cited. cases in which bony fragments have escaped into the articular cavity.

8. In all bilateral fractures of the patella, be they of simultaneous or of successive occurrence.

9. In all refractures, in the absence of contraindications.

10. In old fractures of the patella, associated with marked impairment of function.

In old fractures, two additional steps are added to the usual technic:

1. The resection of the interfragmentary fibrous callus.

2. The freshening of the fractured surfaces.

Heineck operates all compound fractures immediately. In subcutaneous fractures, operative intervention is delayed for from three to five days, being guided by the associated injuries of the soft tissues. Unless contraindications be present, general anesthesia is employed. The employment of the Esmarch bandage is not recommended by the author.

In operating for fractured patella, Heineck generally employs for the exposure of the parts, a flap having its convexity downward. The incision commences on a level with the upper margin of the patella, about one inch to one side, from here it passes downward to a point a little below the apex of the bone, from where it is continued across the limb, and carried to a point corresponding to that from which it started. This incision does not interfere in any way with healing.

Heineck sees no value in the irrigation of the articular cavity, in the waterlogging of the tissues. Only in exceptional cases are the articular cavity or the periarticular tissues drained. The author contents himself of sponging out of the synovial cavity all the extravasated liquid and clotted blood. All completely detached bony fragments either in the articular cavity or in the peri- and paraärticular tissues are removed. For the approximation of the divided soft tissues and for the encircling of the bone, Heineck uses exclusively absorbable ligature and suture material.

Immediately after the operative procedure and the application of the protective dressing to the wound and while the patient is still anesthetised, molded plaster of Paris splint is applied to the injured extremity. This splint should be amply padded, should cover the posterior and lateral surfaces of the limb and should extend from about 10 cm. above the external malleolus to the gluteal fold. The object of this splint is to immobilise the extremity in the position of full extension of the leg on the thigh, and of slight flexion of the thigh on the abdomen. The slight flexion of the thigh on the pelvis has for its purpose the relaxation of the rectus femoris muscle. During the patient's confinement to bed, attention must be given to the heel and to the toes, so as to avoid the development of a pressure-sore upon the former, the heel should be protected by a doughnut pad or other means. By the use of a "cradle" the toes will not be subjected to the weight of the bed clothes and talipes decubitus will not ensue. In the absence of a marked elevation of temperature, of intense pain, of saturation of the dressings, the protective gauze dressings on the joint remain undisturbed for from ten to fifteen days, then, if indicated, the removable sutures are ablated. The immobilising splint is kept in position for about a month.

---

Post-operative gaseous distention of the stomach and intestine can often be relieved by gentle kneading of the abdomen without the necessity of resorting to a stomach or rectal tube.—*International Jour. Surg.*

